# Boosting Synergistic Effects of Short Antimicrobial Peptides With Conventional Antibiotics Against Resistant Bacteria

**DOI:** 10.3389/fmicb.2021.747760

**Published:** 2021-10-18

**Authors:** Chih-Lung Wu, Kuang-Li Peng, Bak-Sau Yip, Ya-Han Chih, Jya-Wei Cheng

**Affiliations:** ^1^Department of Medical Science, Institute of Biotechnology, National Tsing Hua University, Hsinchu, Taiwan; ^2^Department of Neurology, National Taiwan University Hospital Hsinchu Branch, Hsinchu, Taiwan

**Keywords:** antimicrobial peptides (AMP), antibiotic resistance, synergism, bulky non-natural amino acid, end-tagging

## Abstract

The global spread of antibiotic-resistant infections has meant that there is an urgent need to develop new antimicrobial alternatives. In this study, we developed a strategy to boost and/or synergize the activity of conventional antibiotics by combination with antimicrobial peptides tagged with the bulky non-natural amino acid β-naphthylalanine (Nal) to their N- or C-terminus. A checkerboard method was used to evaluate synergistic effects of the parent peptide and the Nal-tagged peptides. Moreover, boron-dipyrro-methene labeled vancomycin was used to characterize the synergistic mechanism of action between the peptides and vancomycin on the bacterial strains. These Nal-tagged antimicrobial peptides also reduced the antibiotic-induced release of lipopolysaccharide from Gram-negative bacteria by more than 99.95%. Our results demonstrate that Nal-tagged peptides could help in developing antimicrobial peptides that not only have enhanced antibacterial activities but also increase the synergistic effects with conventional antibiotics against antibiotic-resistant bacteria.

## Introduction

The emergence of multidrug-resistant pathogens emphasizes the need for novel and alternative therapeutics to fight against infections (Marston et al., 2016). Some AMPs can work synergistically with conventional antibiotics to overcome the resistant problems and reduce the amount of antibiotics (Cassone and Otvos, 2010; Sierra et al., 2017; Martinez et al., 2019; Zharkova et al., 2019; Li et al., 2020). However, rules governing the design of AMPs with synergistic effects are still not clear (Lazar et al., 2018).

Tryptophan, owing to its membrane disruptive and lipid interface anchoring activities, has been found to play important roles in the design and development of Trp-rich AMPs (Mojsoska and Jenssen, 2015; Godballe et al., 2016; Arias et al., 2018). Previously, a Trp-rich peptide PEM-2-W5K/A9W (Ac-KKWRKWLKWLAKK-NH_2_) was developed based on the C-terminal region of *Bothrops asper* myotoxin II (Yu et al., 2010). PEM-2-W5K/A9W was found to possess strong activities against both bacteria and fungi even under high salt conditions (Yu et al., 2010). However, peptide S1 (Ac-KKWRKWLAKK-NH_2_), a shortened version of PEM-2-W5K/A9W, only had antimicrobial activities in the low salt LYM broth media (Chu et al., 2013). The antimicrobial activities of S1 were diminished at high salt concentrations.

Several problems such as salt resistance, proteolytic stability, as well as the lipopolysaccharide (LPS, endotoxin) outer membrane of bacteria may hinder the development of AMPs (Chu et al., 2013; Chih et al., 2015, 2019). Recent studies have indicated that the salt resistance, serum proteolytic stability, and LPS neutralizing activities can be increased by adding bulky non-natural amino acids to the termini of short AMPs such as peptide S1 (Chu et al., 2013; Chih et al., 2015). Nuclear magnetic resonance structures have shown that the two terminal β-naphthylalanine residues of S1-Nal-Nal were inserted into the hydrophobic lipid A motif of LPS micelles and enhanced membrane permeabilization and translocation of this peptide (Yu et al., 2017). Similar results were also observed by adding hydrophobic oligopeptide end tags to the termini of AMPs (Schmidtchen et al., 2009, 2011; Song et al., 2019).

Evidence has highlighted the synergistic effects of using AMPs in combination with conventional antibiotics against multidrug-resistant bacterial strains. For example, polymyxin B and its derivatives were found to function as “permeabilizers” or “potentiators” to sensitize bacteria to other antibiotics or potentiate the action of other antibiotics by interacting with anionic lipopolysaccharide (LPS) outer leaflet of Gram-negative bacteria (Vaara, 2019). It was also shown that multidrug-resistant bacteria frequently show collateral sensitivity to AMPs (Lazar et al., 2018). This finding can be used to develop peptide-antibiotic combinations that can fight against resistant bacteria (Lazar et al., 2018). Zharkova et al. (2019) also found that synergism mainly occurs between highly membrane-active AMPs and antibiotics with intracellular targets. Cytotoxicity of these combinations toward normal eukaryotic cells were rarely synergistic (Zharkova et al., 2019). In addition, a bulky non-natural amino acid substituted version of the antimicrobial peptide Bip-P-113 was found to act as a potentiator to sensitize vancomycin against Gram-negative bacteria such as *Escherichia coli* and to boost synergistic effects with vancomycin against drug-resistant Gram-positive bacteria (Wu et al., 2020).

The aim of this study was to test if the bulky non-natural amino acid end tagging strategy of short AMPs can be used in combination with various antibiotics to fight against antibiotic-resistant bacterial strains. These results could provide important insights into the synergistic mechanism between AMPs and traditional antibiotics.

## Materials and Methods

### Materials

S1, S1-Nal, and S1-Nal-Nal (Table 1) were purchased from Kelowna International Scientific Inc. (Taipei, Taiwan). The identity of the peptides was confirmed by electrospray mass spectroscopy and the purity (>95%) was assessed by high-performance liquid chromatography. Both identity and purity data were provided by Kelowna International Scientific Inc. (Taipei, Taiwan). Tetracycline, ciprofloxacin and vancomycin were purchased from Bio Basic Inc. (Toronto, ON, Canada). BODIPY-labeled Vancomycin was obtained from Thermo Fisher Scientific (Waltham, MA, United States). Mueller-Hinton broth (MHB), Tryptic soy broth (TSB) were purchased from Becton, Dickinson and Company (Franklin Lakes, NJ, United States). Calcein-AM dye was obtained from Sigma-Aldrich (St. Louis, MO, United States).

**TABLE 1:** Sequences of S1, S1-Nal, and S1-Nal-Nal.

**Name**	**Sequence^a^**	**Molecular weight (Da)**
S1	Ac-KKWRKWLAKK-NH2	1,412.79
S1-Nal	Ac-KKWRKWLAKK-Nal-NH2	1,609.98
S1-Nal-Nal	Ac-KKWRKWLAKK-Nal-Nal-NH2	1,806.99

*^a^Nal, β-naphthylalanine.*

### Bacterial Strains and Culture Conditions

*Enterococcus faecium* (BCRC 15B0132, VRE), *Acinetobacter baumannii* strains including 14B0091, 14B0097, and 14B0100, and *E. coli* strains including BCRC 13B0198 and BCRC 13B0207 were purchased from Bioresources Collection & Research Center^1^ (BCRC, FIRDI, Hsinchu, Taiwan). *Escherichia coli* and *A. baumannii* strains were incubated in MHB, and *E. faecium* BCRC 15B0132 were incubated in TSB overnight at 37°C with 150 rpm shaking overnight. The turbidity of bacteria was measured by the absorbance of optical density at 600 nm (OD_600_ = 1, equal to approximately 10^8^ CFU/mL) with UV/Visible spectrophotometer (Biochrom, Cambridge, United Kingdom).

### Antimicrobial Activity Assay

The minimal inhibitory concentrations (MICs) of peptides and antibiotics were determined by using microbroth dilution technique, as described by the guidelines of the Clinical and Laboratory Standards Institute (CLSI) (2015). The assay was conducted manually using the single pipette following the CLSI with some modifications. Briefly, *E. coli* and *A. baumannii* strains were incubated in MHB, and *E. faecium* BCRC 15B0132 were incubated in TSB overnight at 37°C. The cell cultures were regrown to mid-log phase and then diluted to a final concentration of 5 × 10^5^ CFU/mL. 99 μl of each diluted microbes were transferred into each well of a 96-well plate, into which 1 μl of peptides or antibiotics has previously been added and the final concentration of the peptides and antibiotics would be 64–0.13 μg/mL. After incubation for about 16 h at 37°C, the MIC value of peptides or antibiotics was determined by inspecting the visible growth. The MIC value was defined as the lowest concentration of an antimicrobial that will inhibit the visible growth of a microorganism. All experiments were repeated three times independently.

### Checkerboard Assay

The synergistic effects of peptides in combination with antibiotics was assessed by using the microbroth dilution checkerboard assay (Mataraci and Dosler, 2012; Mohamed et al., 2016). Each well containing the mixture of 1 μl peptide (¼ × MIC at final concentration) and 1 μl antibiotic (64–0.13 μg/mL at final concentration), and loaded 98 μl bacteria at the final concentration of 5 × 10^5^ CFU/mL into each well of a 96-well plate. After incubation for about 16 h at 37°C, the MIC value of peptide/antibiotic combination was determined by inspecting the visible growth. MIC values obtained were used to evaluate the effects of combination between peptides and antibiotics by calculating the Fractional Inhibitory Concentration Index (FICI) according to the following formula:


$$\text{FICI}=\frac{{\text{MIC}}_{A}\mathrm{in}\mathrm{combination}}{{\text{MIC}}_{A}\mathrm{alone}}+\frac{{\text{MIC}}_{B}\mathrm{in}\mathrm{combination}}{{\text{MIC}}_{B}\mathrm{alone}}$$

The calculated FICI was interpreted as synergistic (FICI ≤ 0.5), no interaction (0.5 < FICI ≤ 4), or antagonistic (FICI > 4) (Odds, 2003). The experiments were repeated three times independently.

### Confocal Laser Scanning Microscopy

The fluorescence experiments were performed with slight modification as described previously (Joshi et al., 2010). Briefly, the bacteria were diluted to 10^7^ CFU/mL and incubated with S1, S1-Nal, and S1-Nal-Nal peptides at 0.5 × MIC for 30 mins. Then, 2 μg/mL BODIPY-labeled vancomycin were treated for 30 mins. Bacteria treated with BODIPY-labeled vancomycin only served as a control. After treatment, the cell pellet was obtained by centrifugation and washed with PBS to remove free BODIPY-labeled vancomycin. The pellet was resuspended and loaded on the glass slides (Polysine^TM^, Thermo Fisher Scientific, Waltham, MA, United States) and visualized under confocal laser scanning microscope (LSM 510 META, Carl Zeiss, Jena, Thüringen, Germany) equipped with 64 × oil objective lens (Carl Zeiss, Jena, Thüringen, Germany). All experiments were repeated three times independently.

### Calcein Leakage Assay

Calcein acetoxymethyl ester (calcein-AM) is a non-fluorescent dye and enters into the bacteria through diffusing across the cell membrane, and hydrolyzed to fluorescent calcein (C_30_H_26_N_2_O_13_) by cytoplasmic esterases (Xiong et al., 2005). Briefly, the microbes were grown to mid-log phase and the pellet was obtained by centrifugation, washed with PBS, resuspended to OD_600_ of 1.0 with PBS containing 10% (vol/vol) broth. The microbes were incubated with 3 μM calcein-AM for 1 h at 37°C to form calcein-AM loaded cells. The calcein-AM loaded cells were collected by centrifugation (3,000 × *g*, 10 mins), and resuspended to 10^7^ CFU/ml by PBS. Cobalt was added into cells and was used to quench the fluorescence of calcein released into the extracellular environment. The cells were treated with 0.5 × MIC of peptides in the sterile black-wall 96-well plate and measured the fluorescence intensity for 60 mins at an excitation wavelength of 485 nm and an emission wavelength of 510 nm on a fluorescence plate reader (VICTOR3, PerkinElmer, United States) (Essodaigui et al., 1998). Bacteria treated with H_2_O served as negative controls. The intensity of membrane permeabilization (%) was calculated as the absolute percent calcein leakage by peptides with respect to non-treated calcein-AM loaded cells (Koo et al., 2001). All experiments were repeated three times independently.

### Anti-endotoxin Studies

*Escherichia coli* BCRC 13B0198 were cultured at the mid-log phase and diluted to 10^4^ CFU/ml, and then treated with peptide alone at 1 × MIC or combination with vancomycin (both at 0.5 × MIC) at 37°C for 6 h. The cells were filtered by using a pyrogen-free 0.2 μm pore filter (Acrodisc, Pall Corporation, United States) and the endotoxin level was determined by limulus amebocyte lysate (LAL) PYROCHROME^®^ test (Associates of Cape Cod, United States). The kinetic turbidity was analyzed by using a microplate reader (SpectraMax ABS, Molecular Devices, San Jose, CA, United States). The experiments were repeated three times independently.

### Statistical Analysis

The statistical results are performed as the mean ± SEM and were analyzed using one-way ANOVA analysis of variance. Statistical analysis was performed using GraphPad Prism version 8.0 (San Diego, CA, United States), where *p* < 0.05 was considered to indicate a statistically significant difference.

## Results

### Antimicrobial Activity

The antibacterial activities of S1, S1-Nal, S1-Nal-Nal, vancomycin, ciprofloxacin, and tetracycline against Gram-positive bacterium including vancomycin-resistant *E. faecium* BCRC 15B0132 (VRE) and Gram-negative bacteria including *A. baumannii* BCRC 14B0091, *A. baumannii* BCRC 14B0097, *A. baumannii* BCRC 14B0100, *E. coli* BCRC 13B0198, and *E. coli* BCRC 13B0207 were assessed by MIC assay. As shown in Table 2, antibiotics, including vancomycin, ciprofloxacin, and tetracycline had limited or no activities against all bacterial strains except for *E. coli* BCRC 13B0207, which is sensitive to ciprofloxacin. S1 peptide also had limited or no antibacterial activities (>64 μg/ml). On the other hand, peptides S1-Nal and S1-Nal-Nal with the addition of bulky non-natural amino acid β-naphthylalanine at the C-termini demonstrated enhanced activities against all bacterial strains. S1-Nal had the MICs of 64 μg/ml against *E. faecium*, 8 μg/ml against *A. baumannii*, and 32 μg/ml against *E. coli*. S1-Nal-Nal had the MICs of 32 μg/ml against *E. faecium*, 2 μg/ml against *A. baumannii* BCRC 14B0091, 4 μg/ml against *A. baumannii* BCRC 14B0100, 8 μg/ml against *A. baumannii* BCRC 14B0097, and 16 μg/ml against *E. coli*. These results indicated that the addition of only one or two bulky non-natural amino acid end tags could boost the antimicrobial activity.

**TABLE 2:** The minimal inhibitory concentrations (MICs) of S1, S1-Nal, S1-Nal-Nal, vancomycin, ciprofloxacin, and tetracycline.

**Bacterial strains**	**MIC^a^ (μg/ml)**					
	**S1**	**S1-Nal**	**S1-Nal-Nal**	**Vancomycin**	**Ciprofloxacin**	**Tetracycline**
*Enterococcus faecium* BCRC 15B0132	>64	64	32	>64	>64	64
*Acinetobacter baumannii* BCRC 14B0091	>64	8	2	>64	32	>64
*Acinetobacter baumannii* BCRC 14B0097	>64	8	8	>64	>64	>64
*Acinetobacter baumannii* BCRC 14B0100	>64	8	4	>64	64	>64
*Escherichia coli* BCRC 13B0198	64	32	16	>64	32	>64
*Escherichia coli* BCRC 13B0207	>64	32	16	>64	0.25	>64

*^a^MIC, minimum inhibitory concentration.*

### Bacterial Membrane Permeabilization

**FIGURE 1:**
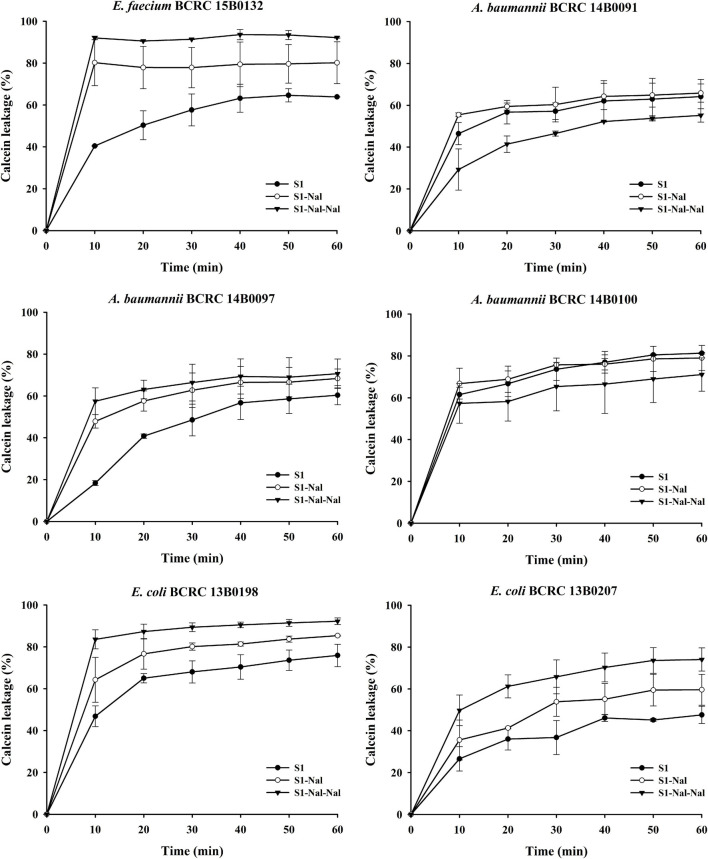
The membrane permeabilization of *Enterococcus faecium* BCRC 15B0132, *Acinetobacter baumannii* BCRC 14B0091, *A. baumannii* BCRC 14B0097, *A. baumannii* BCRC 14B0100, *Escherichia coli* BCRC 13B0198, and *E. coli* BCRC 13B0207, by S1, S1-Nal, and S1-Nal-Nal for 60 min exposure. Calcein-AM loaded cells (10^7^ CFU/ml) were resuspended by PBS, and the aliquots of 100 μl were added into a sterile black-wall 96-well plate, then treated with 0.5 × MIC of peptides in each well and measured the calcein leakage immediately. Each time point for the peptides was repeated three times independently.

Permeabilities of S1, S1-Nal, and S1-Nal-Nal were measured by the peptide-induced leakage of the fluorescent dye calcein from bacterial cells (Figure 1). To determine the mechanism of antibacterial action of the AMPs, we evaluated the ability of peptides to permeabilize intact *E. faecium*, *A. baumannii*, and *E. coli* membranes by measuring the peptide-induced leakage of the fluorescent dye calcein from bacterial cells (Figure 1). In general, the permeabilization ability is in the order of S1-Nal-Nal > S1-Nal > S1. Addition of β-naphthylalanine to the termini of S1-Nal and S1-Nal-Nal can increase permeability from 60 to 90% for *E. faecium*, from 50 to 60% for *A. baumannii* BCRC 14B0097, from 65 to 90% for *E. coli* BCRC13B0198, and from 40 to 70% for *E. coli* BCRC13B0207. However, for bacterial strains *A. baumannii* BCRC 14B0091 and *A. baumannii* BCRC 14B0100, the order of the permeabilization ability is S1-Nal = S1 > S1-Nal-Nal. Nevertheless, All peptides showed substantial release of calcein from these six bacterial cells.

### Synergistic Effect With Vancomycin, Ciprofloxacin, and Tetracycline in the Presence of a Sub-Inhibitory Concentration (¼ × Minimal Inhibitory Concentrations) of Peptides

Based on the strong antimicrobial activity and bacterial membrane permeability, it is suggested that these peptides could be used to potentiate conventional antibiotics such as vancomycin, ciprofloxacin, and tetracycline against antibiotic-resistant Gram-positive and Gram-negative bacterial strains. Synergetic activities of S1, S1-Nal, and S1-Nal-Nal combined with vancomycin, ciprofloxacin, and tetracycline were then determined by the checkerboard assay (Table 3). For *E. faecium* BCRC 15B0132 and *A. baumannii* BCRC 14B0097, S1-Nal-Nal showed substantial synergy while combined with vancomycin, ciprofloxacin, and tetracycline. The order of the synergistic effects against *E. faecium* BCRC 15B0132 and *A. baumannii* BCRC 14B0097 is S1-Nal-Nal > S1-Nal > S1. For *A. baumannii* BCRC 14B0091 and *A. baumannii* BCRC 14B0100, S1-Nal demonstrated better synergistic effects than S1-Nal-Nal and S1. For *E. coli* BCRC 13B0198, all peptides had strong synergy while combined with ciprofloxacin and vancomycin, but these three peptides had only limited or no synergy while combined with tetracycline. For *E. coli* BCRC 13B0207, all peptides demonstrated strong synergy while combined with ciprofloxacin, but had only limited activity while combined with vancomycin and tetracycline.

**TABLE 3:** Synergistic effects of S1, S1-Nal, and S1-Nal-Nal in combination with antibiotics against bacterial strains studied.

**Strains**	**Antibiotics (μg/mL)**	**AMP (μg/mL) (¼ × MIC)**					
		**S1**		**S1-Nal**		**S1-Nal-Nal**	
		**+**	**FICI^a^**	**+**	**FICI**	**+**	**FICI**
*Enterococcus faecium* BCRC 15B0132	Vancomycin	>64	1.25	8	**0.31**	2	**0.27**
	Ciprofloxacin	64	0.75	16	**0.38**	2	**0.27**
	Tetracycline	16	**0.5**	8	**0.38**	1	**0.27**
*Acinetobacter baumannii* BCRC 14B0091	Vancomycin	16	**0.38**	2	**0.27**	>64	1.25
	Ciprofloxacin	32	**0.5**	32	**0.5**	64	2.25
	Tetracycline	64	0.75	32	**0.5**	>64	1.25
*Acinetobacter baumannii* BCRC 14B0097	Vancomycin	32	**0.5**	2	**0.27**	2	**0.27**
	Ciprofloxacin	>64	1.25	64	0.75	2	**0.27**
	Tetracycline	64	0.75	64	0.75	2	**0.27**
*Acinetobacter baumannii* BCRC 14B0100	Vancomycin	32	**0.5**	2	**0.27**	4	**0.28**
	Ciprofloxacin	64	0.75	32	**0.5**	32	0.75
	Tetracycline	64	0.75	64	0.75	>64	1.25
*Escherichia coli* BCRC 13B0198	Vancomycin	32	**0.5**	32	**0.5**	32	**0.5**
	Ciprofloxacin	2	**0.27**	2	**0.27**	2	**0.31**
	Tetracycline	>64	1.25	64	0.75	64	0.75
*Escherichia coli* BCRC 13B0207	Vancomycin	64	0.75	>64	1.25	64	0.75
	Ciprofloxacin	0.02	**0.31**	0.06	**0.5**	0.13	0.75
	Tetracycline	64	0.75	64	0.75	64	0.75

*^a^FICI, fractional inhibitory concentration index, FICI ≤ 0.5, synergy; 0.5 < FICI ≤ 4, no interaction; FICI > 4, antagonism.*

*+, the MIC of antibiotic in combination with ¼ × MIC antimicrobial peptides (AMPs). Bold values indicated synergistic effects.*

### Mechanism of the Synergistic Effects Studied by Boron-Dipyrro-Methene Labeled Vancomycin

**FIGURE 2:**
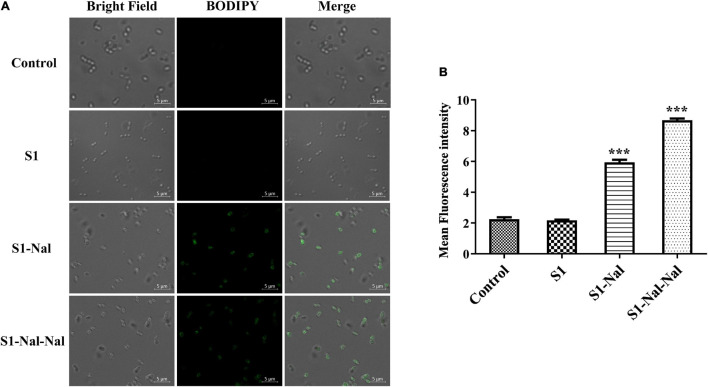
S-Nal and S1-Nal-Nal increase the uptake of boron-dipyrro-methene (BODIPY)-labeled vancomycin in *E. faecium*. **(A)** Fluorescence images of 10^7^ CFU/ml *E. faecium* BCRC 15B0132 treated with 0.5 × MIC of S1, S1-Nal and S1-Nal-Nal at 37°C for 30 min, then treated BODIPY-labeled vancomycin for 30 min (scale bar represents 5 μm). **(B)** Mean fluorescence intensity of S1, S1-Nal, and S1-Nal-Nal treated in *E. faecium* BCRC 15B0132. Samples treated with BODIPY-labeled vancomycin only served as a control. Results are presented as means ± SEM, ***P < 0.001 compared with control. n.s., no significant differences.

**FIGURE 3:**
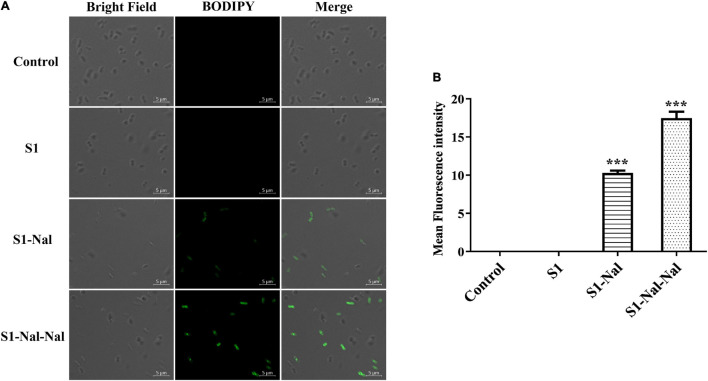
S1-Nal and S1-Nal-Nal increase the uptake of BODIPY-labeled vancomycin in *A. baumannii*. **(A)** Fluorescence images of 10^7^ CFU/ml *A. baumannii* BCRC 14B0097 treated with 0.5 × MIC of S1, S1-Nal, and S1-Nal-Nal at 37°C for 30 min, then treated BODIPY-labeled vancomycin for 30 min (scale bar represents 5 μm). **(B)** Mean fluorescence intensity of S1, S1-Nal, and S1-Nal-Nal treated in *A. baumannii* BCRC 14B0097. Samples treated with BODIPY-labeled vancomycin only served as a control. Results are presented as means ± SEM, ***P < 0.001 compared with control. n.s., no significant differences.

**FIGURE 4:**
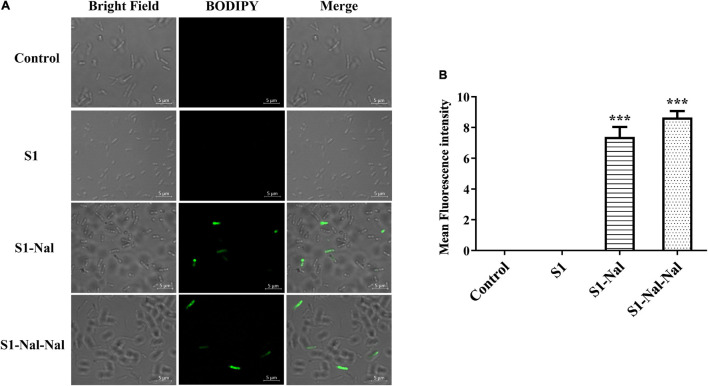
S1-Nal and S1-Nal-Nal increase the uptake of BODIPY-labeled vancomycin in *E. coli*. **(A)** Fluorescence images of 10^7^ CFU/ml *E. coli* BCRC 13B0207 treated with 0.5 × MIC of S1, S1-Nal, and S1-Nal-Nal at 37°C for 30 min, then treated BODIPY-labeled vancomycin for 30 mins (scale bar represents 5 μm). **(B)** Mean fluorescence intensity of S1, S1-Nal, and S1-Nal-Nal treated in *E. coli* BCRC 13B0207. Samples treated with BODIPY-labeled vancomycin only served as a control. Results are presented as means ± SEM, ***P < 0.001 compared with control.

Boron-Dipyrro-Methene (BODIPY)-labeled vancomycin was used to study the mechanism of synergistic effects of S1, S1-Nal, and S1-Nal-Nal by measuring the uptake of vancomycin in *E. faecium* BCRC 15B0132, *A. baumannii* BCRC 14B0097, and *E. coli* BCRC 13B0207 strains (Chu et al., 2020). The results demonstrated that S1-Nal and S1-Nal-Nal with β-naphthylalanine end-tagging markedly enhanced the entry of BODIPY-labeled vancomycin into the Gram-positive *E. faecium* BCRC 15B0132 strain (Figure 2). Similar results were also found for the Gram-negative *A. baumannii* BCRC 14B0097, and *E. coli* BCRC 13B0207 strains (Figures 3, 4).

### S1-Nal and S1-Nal-Nal Attenuate Vancomycin-Induced Lipopolysaccharide Release

**FIGURE 5:**
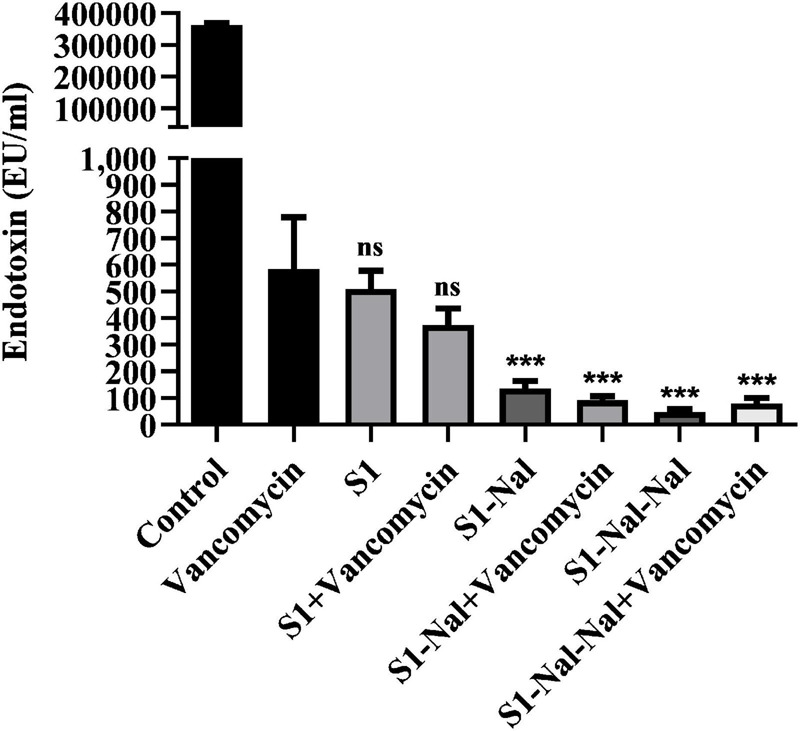
Attenuation of LPS release from *E. coli*. *Escherichia coli* BCRC 13B0198 were incubated at the mid-log phase (10^4^ CFU/ml) and treated with peptide alone (at 1 × MIC) or combination with vancomycin (both at 0.5 × MIC) at 37°C for 6 h. The samples were filtered through 0.2 μm pore filter and the endotoxin level was detected by LAL assay. ***P < 0.001 compared with vancomycin only. ns, no significant differences compared with vancomycin only.

Antibiotic-treatment can cause the release of lipopolysaccharide (LPS, endotoxin) from Gram-negative bacteria into the bloodstream of the host and has been shown to be associated with the deterioration of the patients (Trautmann et al., 1998; Li et al., 2017). Herein, we used LAL assay to measure the concentration of LPS in supernatants induced by the treatment of vancomycin (Figure 5). *Escherichia coli* BCRC 13B0198 cells were treated with S1, S1-Nal, and S1-Nal-Nal or the combination of vancomycin and the peptides. The LPS concentration decreased tremendously with the S1-Nal, and S1-Nal-Nal treatment alone or the combination of S1-Nal, S1-Nal-Nal, and vancomycin treatment (Figure 5).

## Discussion

End-tagging peptides with hydrophobic moieties have been developed to increase salt resistance and potency of AMPs. For example, tryptophan and/or phenylalanine stretch (Schmidtchen et al., 2009), as well as fatty acid, vitamin E, or cholesterol were added to the termini of short AMPs (Avrahami and Shai, 2002; Makovitzki et al., 2006; Serrano et al., 2009; Rosenfeld et al., 2010; Arnusch et al., 2012). This strategy was modified by adding only one or two β-naphthylalanine to the termini of short antimicrobial peptide to boost its salt resistance, serum proteolytic stability and antiendotoxin activities (Chu et al., 2013; Chih et al., 2015). There are several advantages to using β-naphthylalanine than using tryptophan/phenylalanine or fatty acid as end-tags. For example, a stretch of five tryptophan end-tags were needed to provide salt resistance to the antimicrobial peptide KNK10 under 150 mM NaCl (Pasupuleti et al., 2009). Only one β-naphthylalanine end-tag was needed to provide substantial salt resistance (Chu et al., 2013). Furthermore, β-naphthylalanine end-tags can provide superior serum stability due to its non-natural and bulky characteristics (Chu et al., 2013; Chih et al., 2015). This is particularly important for the development of short AMPs to lower the cost of synthesis. In this study, we have extended the β-naphthylalanine end-tagging strategy to boost the synergistic effects of S1-Nal and S1-Nal-Nal with conventional antibiotics against drug-resistant bacteria. Our results indicated that both S1-Nal and S1-Nal-Nal displayed synergistic effects in combination with conventional antibiotics such as vancomycin, ciprofloxacin, and tetracycline against the Gram-positive *E. faecium* BCRC15B0132 strain. S1-Nal or S1-Nal-Nal also displayed synergistic effects with vancomycin and ciprofloxacin against the Gram-negative *A. baumannii* BCRC14B0091, *A. baumannii* BCRC14B0097, *A. baumannii* BCRC14B0100, *E. coli* BCRC13B0198, and *E. coli* BCRC13B0207 strains. However, lack of synergy with tetracycline for all peptides was found against the *A. baumannii* BCRC14B0100, *E. coli* BCRC13B0198, and *E. coli* BCRC13B0207 strains.

Polymyxin B has been used as a potentiator to act synergistically with antibiotics against drug-resistant Gram-negative bacteria (Vaara, 2019). The mechanism of synergism was attributed to the increase of bacterial membrane permeabilization caused by polymyxin B (Vaara, 2019). AMP DP7 shows synergistic effects with the antibiotic, azithromycin, against the antibiotic-resistant strains including *S. aureus* and *P. aeruginosa* isolates (Wu et al., 2017). The synergistic effects may be attributed to the cell wall proteins reduced by azithromycin and cell wall disruption by DP7 peptide (Wu et al., 2017). Antimicrobial peptide CLP-19 displayed synergistic effects with antibiotics such as ampicillin, ceftazidime, and levofloxacin against *E. coli* ATCC 25922 and *S. aureus* ATCC 29213 (Li et al., 2017). In addition, the CLP-19 and CLP-19/antibiotic combination could induce the generation of hydroxyl radicals from bacteria through depleted NADH, which were related to the synergistic effects of CLP-19/antibiotic combination (Li et al., 2017). As can be seen from Figure 1 in this study, increases of membrane permeabilization of β-naphthylalanine end-tagging against the six bacterial strains studied were found. The results demonstrated that the increase of membrane permeabilization correlated well with synergistic effect with antibiotics against drug-resistant bacterial strains. Moreover, BODIPY-labeled vancomycin was used to study membrane permeabilization and uptake of vancomycin in *E. faecium*, *A. baumannii*, and *E. coli* strains (Figures 2–4). The results indicated that the antimicrobial peptide-induced fluorescent intensity changes of BODIPY-labeled vancomycin inside bacterial cells were concordant with the membrane permeability of the peptides. One interesting thing to note from bacterial membrane permeabilization studies is that β-naphthylalanine end-tags caused higher membrane permeabilization of the Gram-positive bacterial strain (from 60% calcein leakage of S1 to 90% calcein leakage of S1-Nal-Nal) than the Gram-negative bacterial strains (Figure 1). S1-Nal and S1-Nal-Nal both have excellent synergistic effects with vancomycin, ciprofloxacin, and tetracycline against the Gram-positive *E. faecium* BCRC 15B0132 strain (Table 3).

Lipopolysaccharide constitutes the major component of the outer leaflet of Gram-negative bacteria. It was shown that S1-Nal and S1-Nal-Nal possess high antiendotoxin activities (Chih et al., 2015). Furthermore, S1-Nal and S1-Nal-Nal can inhibit LPS-induced nitrite oxide and TNF-α production in murine macrophage cells and suppress TNF-α release in endotoxemia mouse model (Chih et al., 2015). NMR structural studies of S1-Nal-Nal and LPS micelles complex indicated that S1-Nal-Nal rotated its two terminal β-naphthylalanine residues into the hydrophobic lipid A motif of LPS micelles and blocked the LPS-induced inflammation (Yu et al., 2013). We and others have demonstrated that some AMPs may reduce antibiotic-induced release of LPS from Gram-negative bacteria (Li et al., 2017; Chu et al., 2020). In this study, we have found that S1-Nal and S1-Nal-Nal can reduce antibiotic-induced release of LPS from *E. coli* (Figure 5).

**FIGURE 6:**
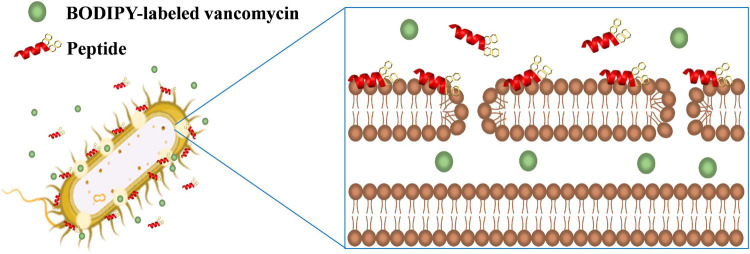
Schematic illustration of the synergistic mechanism for BODIPY-labeled vancomycin and peptides. S1-Nal-Nal (red) disturbs the bacterial outer membrane and enhances the entry of BODIPY-labeled vancomycin (green circle) into bacteria.

Vancomycin was shown to act synergistically with AMPs against vancomycin-persistent Gram-positive bacterial cells (Feng et al., 2015). However, vancomycin itself has no antibacterial effect against Gram-negative bacteria due to the LPS outer leaflet preventing large glycopeptide antibiotics such as vancomycin from being transported to intracellular targets. Recently, it was found that vancomycin can eradicate some *E. coli* cells under cold stress conditions through inhibition of peptidoglycan biosynthesis that is similar to the mechanism of action of vancomycin to Gram-positive bacteria (Stokes et al., 2016). Silver ion was also shown to increase membrane permeability of *E. coli* cells and potentiate vancomycin against *E. coli* (Morones-Ramirez et al., 2013). In this study, we have also demonstrated that the β-naphthylalanine end-tagged S1-Nal and S-Nal-Nal can be used with vancomycin to fight against Gram-negative bacterial infections (Figure 6).

In conclusion, we describe a strategy to boost synergistic effects of short AMPs with conventional antibiotics against resistant bacterial strains by adding β-naphthylalanine (Nal) to one of the peptides’ termini. The described Nal-tagged peptides (S1-Nal and S1-Nal-Nal) enhanced the antibacterial activity of the plain peptides and generated a better synergistic effect when combined with conventional antibiotics. Increasing the membrane permeabilization against the bacterial strains was also observed. Additionally, these Nal-tagged AMPs reduced the antibiotic-induced release of LPS from Gram-negative bacteria by more than 99.95%. Only one or two Nal end-tags were needed to have these biological impacts on the peptides, making this a potential strategy for developing new antimicrobial agents.

## Data Availability Statement

The original contributions presented in the study are included in the article/supplementary material, further inquiries can be directed to the corresponding author.

## Author Contributions

C-LW, Y-HC, and K-LP performed the experiments and analyzed the data. C-LW and J-WC wrote the manuscript. B-SY and J-WC planned the study and revised the manuscript. All authors contributed to the article and approved the submitted version.

## Conflict of Interest

The authors declare that the research was conducted in the absence of any commercial or financial relationships that could be construed as a potential conflict of interest.

## Publisher’s Note

All claims expressed in this article are solely those of the authors and do not necessarily represent those of their affiliated organizations, or those of the publisher, the editors and the reviewers. Any product that may be evaluated in this article, or claim that may be made by its manufacturer, is not guaranteed or endorsed by the publisher.

## Footnotes

1
https://catalog.bcrc.firdi.org.tw/
